# Defining the benefit: Cardioversion of persistent atrial fibrillation

**DOI:** 10.1016/j.ijcha.2021.100882

**Published:** 2021-09-30

**Authors:** Sotirios Nedios, David Duncker

**Affiliations:** Department of Electrophysiology, Heart Center Leipzig, Leipzig, Germany; Hannover Heart Rhythm Center, Department of Cardiology and Angiology, Hannover Medical School, Hannover, Germany

**How do you find out whether rhythm control is the right strategy for your patient?**

Atrial fibrillation (AF) is a disease of advanced age and thus more common in an ageing population [1]. Certainly, the primary concern is to prevent complications like stroke, debilitating symptoms, heart failure or hospitalizations. A number of people though do not notice the subtle change of settling AF, unless they come back to sinus rhythm (SR). Thus, for many years rhythm control was considered a preferable first attempt and rate control was largely seen as a fallback option. However, AF recurrence is common and maintaining SR is not easy. Therefore, studies that help us identify patients that could profit from a sustained rhythm control strategy could improve our clinical practice.

In this issue of the International Journal of Cardiology Heart & Vasculature, Hermans et al. [2] report on the clinical utility of electrical cardioversion (ECV) for the evaluation of symptom-rhythm correlation (SRC), defined as self-reported relief during sinus rhythm. The authors examined this rhythm control strategy in 81 persistent AF patients presenting with dyspnea (46%), exercise intolerance (20%), tiredness (6%), palpitations (5%), chest pain (1%) or no symptoms at all (22%). Interestingly, at one month after ECV only one out of three remained in SR (34%) and only one out of five reported a SRC improvement (22%). In contrast, the majority had no (35%) or un-evaluable (43%) SRC. These patients usually had more AF recurrence and reported different symptom patterns than upon presentation. Given the low yield, the authors conclude that cardioversion alone often results in symptom changes, but rarely in symptom-rhythm correlation.

This study provides some interesting insights that question the utility of ECV for symptom-rhythm correlation. In contrast to catheter ablation, ECV did not result in a significant symptom relief [3]. Certainly, every physician interaction may have a placebo effect or may help address cardiovascular factors that contribute to symptom burden. As shown in this study though, a clear improvement comes only by sustaining sinus rhythm. Since AF recurrence after ECV is common, more studies are warranted to identify the optimal strategy that differentiates “AF symptoms” from “symptoms in AF”.

Hermans et al. are commended for evaluating the concept of ECV with a later outpatient follow-up [2]. Despite the inherent limitations of a retrospective study, some possible selection bias and the lack of continuous monitoring for AF recurrence, they found that this strategy is not very helpful. In contrast, the EAST-AFNET 4 trial showed that a rhythm-control strategy is superior to rate control in improving cardiovascular outcomes (esp. death or stroke), even for asymptomatic patients with recent AF diagnosis [4]. In light of these results, one might well ask whether we should abandon SRC with an ECV trial and go directly for an ablation.

In the last decades, several major rate versus rhythm control studies (AFFIRM, RACE, AF-CHF) came to the same conclusion: rhythm control was not superior in terms of morbidity or mortality and rate control was a legitimate primary treatment option [5], [6], [7]. These studies though included patients with more sustained AF than the EAST-AFNET trial (within 12 months of diagnosis) and did not account for self-reported symptoms. Thus, the benefit seen with an early treatment may not be evident in more advanced stages and further research is needed to evaluate the effect of rhythm control on patient reported outcomes for persistent AF [8].

Similar to previous studies the study by Hermans et al. confirms that SR correlates with relief, but AF recurrence after ECV is common in > 50% of the patients [9]. This shows that the issue is not whether SR is better, but rather that it is difficult to maintain. It’s important to consider that AF recurrence is not treatment failure per se and that better patient selection (e.g. early stages, smaller atria) and additional therapy (antiarrhythmic drugs/ablation) may improve the SRC and the treatment of these patients. In short, don’t lose time on a single ECV, but rather consider the chances of AF recurrence and act appropriately. Furthermore, patient education on symptom development and quality is needed in order to better assess symptom severity [10].

Hermans et al. [2] are congratulated for their study. Their results challenge the idea of ECV as a tool for symptom-rhythm correlation and highlight the need for improved rhythm-control strategies. The advantage of rhythm over rate control for cardiovascular outcomes in persistent AF is not clear and thus patient-reported improvement remains critical for the escalation of therapy. The increasing evidence for benefits of AF screening in an aging population may result in a surge of oligo- or asymptomatic persistent AF patients. Identifying those who will profit from rhythm control is crucial for applying therapeutic interventions while respecting health care resources. Surely, mobile health-based (MHealth) and individualized solutions may help reach this goal [11]. Since a cardioversion with an outpatient follow-up has low yield though, better strategies are needed to sustain SR and assess its benefit in patients with persistent AF. In that sense, more studies are encouraged to explore and guide the management of these patients.
